# Pigmented paravenous retinochoroidal atrophy: a case report

**DOI:** 10.1186/s12886-018-0809-z

**Published:** 2018-06-07

**Authors:** Yinchen Shen, Xun Xu, Hui Cao

**Affiliations:** Department of Ophthalmology, Shanghai General Hospital, Shanghai Jiao Tong University School of Medicine (originally named “Shanghai First People’s Hospital”), No. 100 Haining Road, Shanghai, 200080 People’s Republic of China

**Keywords:** Pigmented paravenous retinochoroidal atrophy, Fundus autofluorescence, Spectral domain optical coherence tomography, Optical coherence tomography angiography, Electroretinogram

## Abstract

**Background:**

Pigmented paravenous retinochoroidal atrophy (PPRCA) is an unusual retinal degeneration, and its performance on optical coherence tomography angiography (OCTA) is unclear. We report a Chinese female case of PPRCA and her OCTA features.

**Case presentation:**

A 66-year-old female patient was referred to the author’s center for gradual progressive loss of vision in both eyes and photophobia of 2 years duration. She reported having no family history of inherited ocular diseases. The funduscopic examination revealed bone-spicule pigmentation and retinochoroidal atrophy along the retinal veins. This patient was diagnosed with PPRCA which is a rare disease, uncommon in females, more commonly affecting the paravascular fundus. Noninvasive imaging techniques features of this patient was described, including ultra-wide field fundus autofluorescence, spectral domain optical coherence tomography (SD-OCT), OCTA (SSADA), etc. The en face OCTA images demonstrated areas of flow void beneath the retinal pigment epithelium-Bruch membrane layer suggestive of choriocapillaris hypoperfusion that corresponded with indocyanine green angiography (ICGA). Further studies should be conducted to clarify the relationship between choriocapillaris hypoperfusion and the development of PPRCA.

**Conclusions:**

The OCTA features in patients with PPRCA has not been described previously in the literature. This case might provide preliminary information regarding the pathophysiology of PPRCA and improve our understanding of the nature of this disease.

## Background

Pigmented paravenous retinochoroidal atrophy (PPRCA) is an unusual retinal degeneration characterized by perivenous aggregations of pigment clumps associated with peripapillary and radial zones of retinochoroidal atrophy that are distributed along the retinal veins [1]. Patients are often asymptomatic and the diagnosis is based on a characteristic fundus appearance [2]. The etiology remains unknown or idiopathic, although many report of familial cases make hereditary nature of this condition quite apparent [3]. We here report a Chinese female case of PPRCA.

## Case presentation

A 66-year-old female patient was referred to the authors’ center for gradual progressive loss of vision in both eyes and photophobia of 2 years duration. She reported having no family history of inherited ocular diseases, no history of trauma, no history of inflammation or infectious diseases. Her best corrected visual acuity (BCVA) was 65 ETDRS letters (Snellen equivalent 20/50) in the right eye and 61 ETDRS letters (Snellen equivalent 20/62.5) in the left eye. The anterior segment was unremarkable in both eyes. No relative afferent pupillary defect was found. The intraocular pressure was normal. Funduscopic examination after pupillary dilation found bone-spicule pigmentation and retinochoroidal atrophy along the retinal veins, bilaterally, without macular involvement or signs of inflammation.

**Fig. 1:**
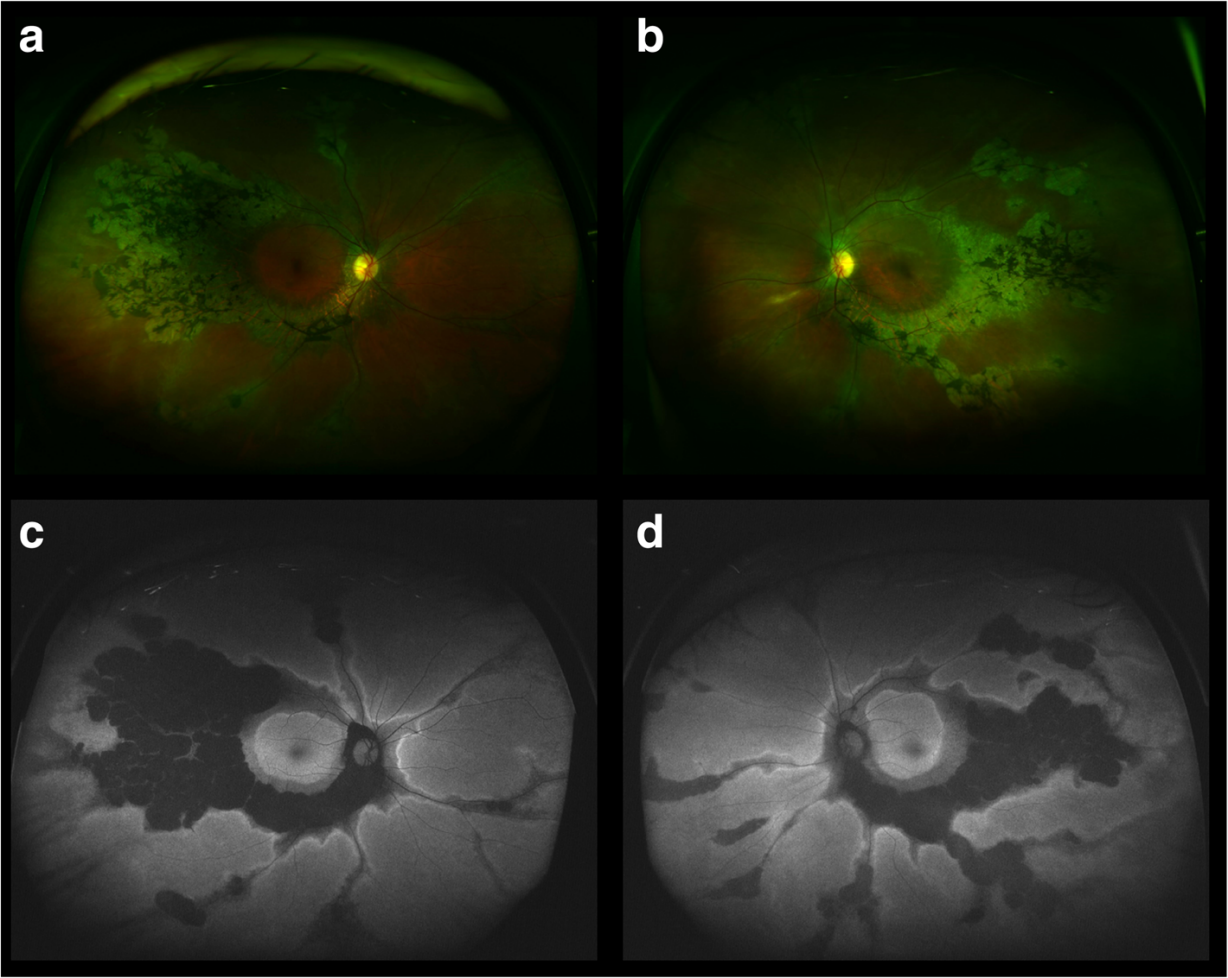
Ultra-wide field fundu photography showed bone-spicule pigmentation and retinochoroidal atrophy in the peripheral paravenous distribution, bilaterally, without macular involvement (right eye **a**; left eye **b**). Fundus autofluorescence imaging revealed sharply demarcated hypoautofluorescent areas corresponding to the atrophic patches along the vein, surrounded by a relatively hyperautofluorescent band (right eye **c**; left eye **d**)

**Fig. 2:**
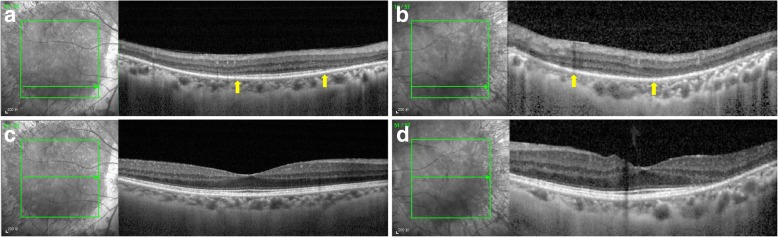
Spectral domain optical coherence tomography (SD-OCT) imaging found an obvious thinning of the entire outer retina (yellow arrow) including outer nuclear layer, external limiting membrane, myoid zone, ellipsoid zone, and interdigitation zone in the paravenous areas (right eye **a**; left eye **b**), but the microstructure of the macular remained intact (right eye **c**; left eye **d**)

**Fig. 3:**
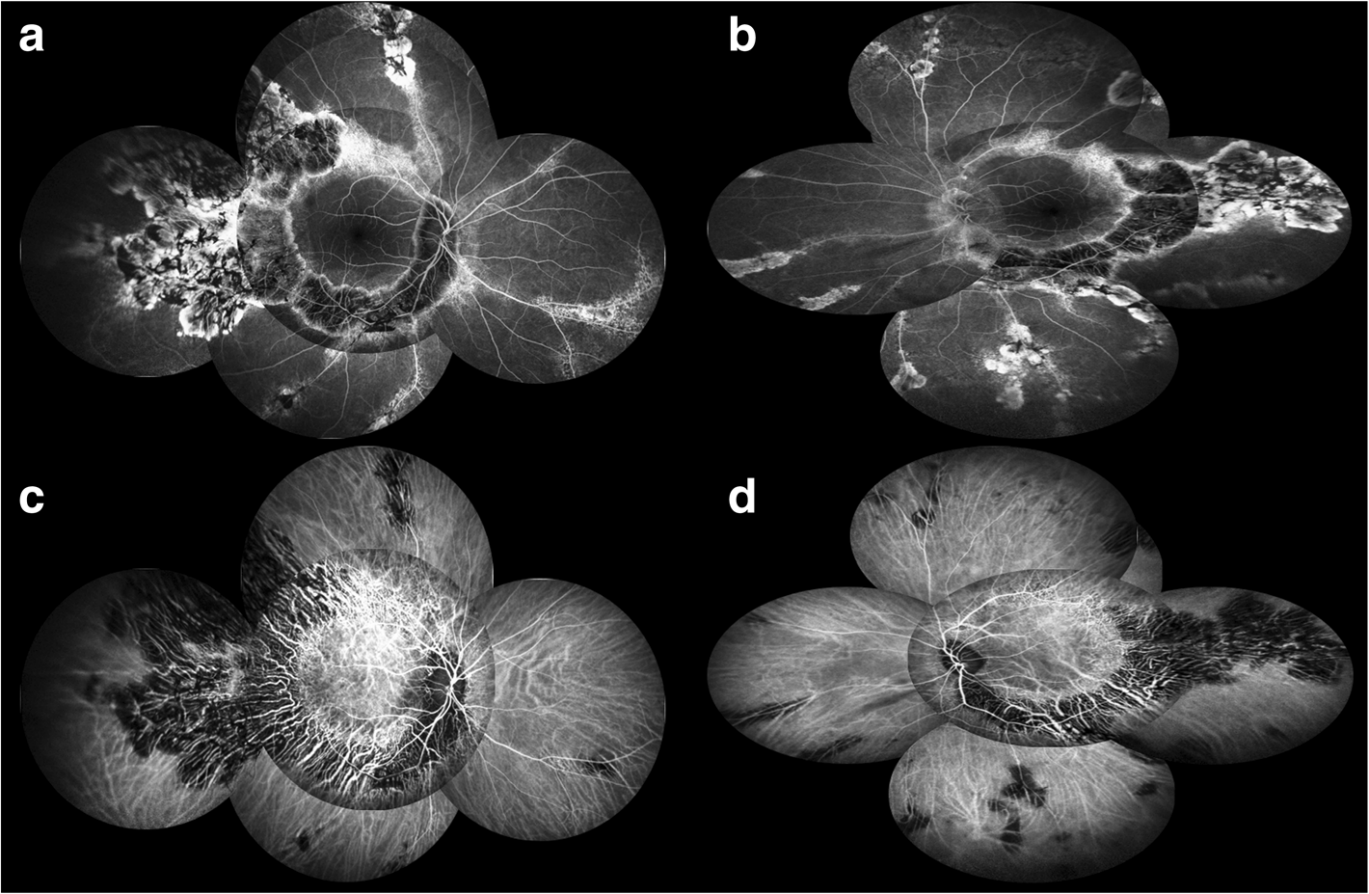
Fluorescein angiography (FA) and indocyanine green angiography (ICGA) showed a window defect (**a, b**) with visualization of medium-to-large-caliber choriocapillary vessels and hypofluorescence (**c, d**), respectively, corresponding to the atrophic area along the veins and the optic disc. FA also show staining along borders of atrophic areas

**Fig. 4:**
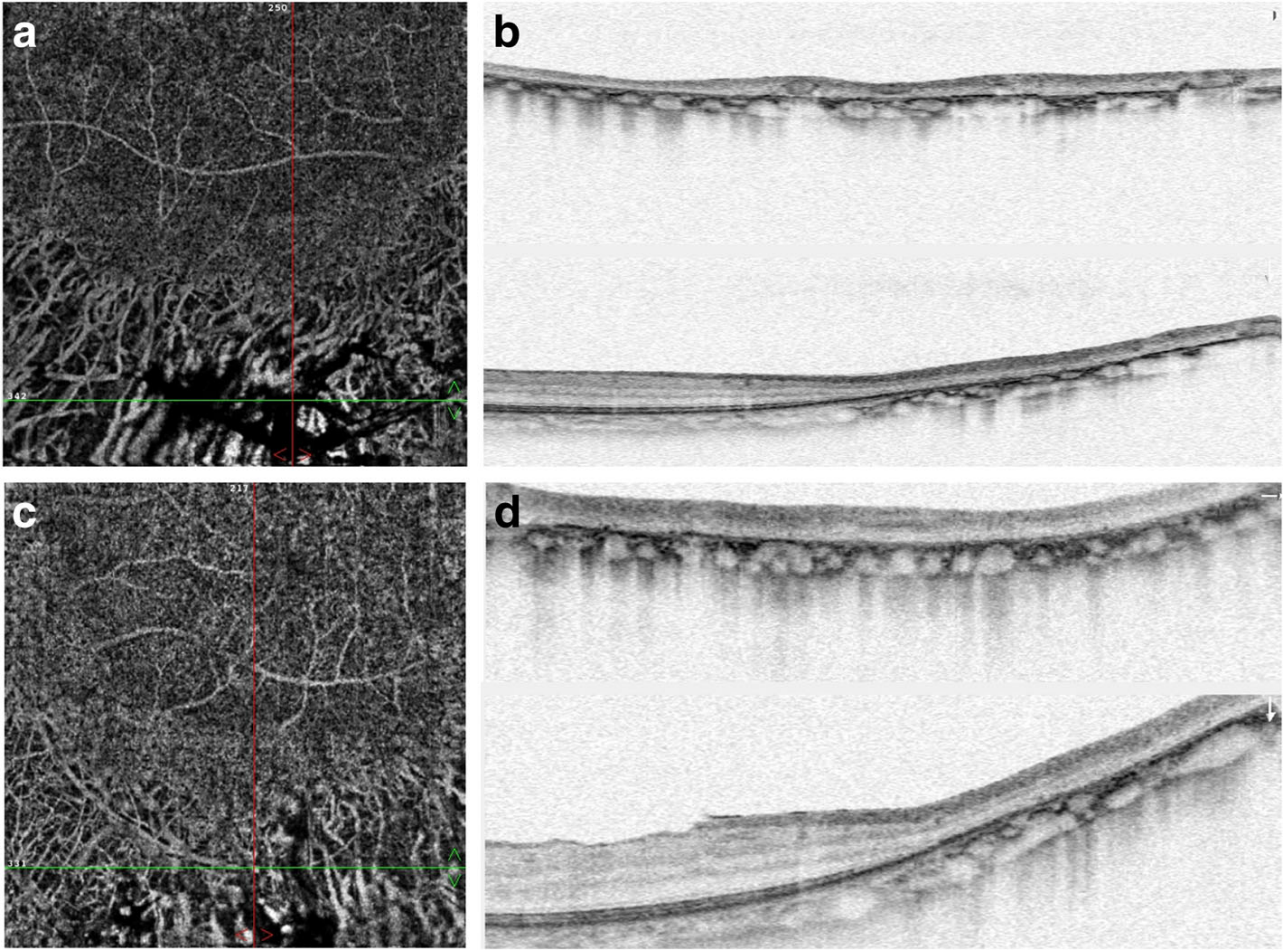
Within the choroid capillary layer, the en face OCTA images demonstrated areas of flow void beneath the retinal pigment epithelium-Bruch membrane layer suggestive of choriocapillaris hypoperfusion that corresponded with ICGA, and there also appear to be more large-caliber vessels in areas of choriocapillary loss (**a** and **c** were the en face OCTA images within the choroid capillary layer for the right eye and the left eye, respectively; **b** and **d** were the horizontal and vertical OCT scans for the right eye and the left eye, respectively)

Ultra-wide field fundus autofluorescence imaging revealed demarcated hypoautofluorescent areas corresponding to the atrophic patches in the peripheral paravenous distribution, surrounded by a relatively hyperautofluorescent band (Fig. 1). Spectral domain optical coherence tomography (SD-OCT) imaging found an obvious thinning of the entire outer retina, but the microstructure of the macular remained intact (Fig. 2). Fluorescein angiography (FA) and indocyanine green angiography (ICGA) showed a window defect with visualization of medium-to-large-caliber choriocapillary vessels and hypofluorescence, respectively (Fig. 3), corresponding to the atrophic area along the veins and the optic disc. FA also show staining along borders of atrophic areas. In order to better reveal the subtle abnormalities that may not be detectable on routine FA and ICGA, 6*6 mm optical coherence tomography angiography (OCTA, SSADA) images were acquired to capture more information. Within the choroid capillary layer, the en face OCTA images demonstrated areas of flow void beneath the retinal pigment epithelium-Bruch membrane layer suggestive of choriocapillaris hypoperfusion that corresponded with ICGA, and there also appear to be more large-caliber vessels in areas of choriocapillary loss. (Fig. 4). Electroretinogram (ERG) of both eyes showed mildly subnormal responses. A diagnosis of PPRCA was arrived at. The nature of the disease its prognostic was explained to the patient.

## Discussion

PPRCA is a rare ocular disease with unknown etiology and slow progression. For this patient, the cause of the disease still remains unclear. Although the diagnosis of PPRCA is based on typical fundus appearance, the findings of fundus autofluorescence, FA, ICGA, and ERG can help us to confirm the diagnosis.

In this case, OCTA is applied to analyze the vascular structures of the retina and the choroid to reveal subtle abnormalities. OCTA is a novel imaging technique that provides noninvasive angiographic maps of the retinal and choroidal vasculature. The technique of OCTA is based on split-spectrum amplitude-decorrelation angiography that is able to detect endoluminal flow and reconstruct the retinochoroidal microvascular network [4]. OCTA images of our patient demonstrated areas of flow void suggestive of choriocapillaris hypoperfusion, which may lead to insufficient metabolic supplementation for the outer retinal structures [5], but hypoperfusion of the choriocapillars could also result from RPE/ outer retinal loss. Therefore, it is unclear whether choroidal blood flow diminution is a etiology or a coexisting finding in this case. Further studies should be conducted to clarify the relationship between choriocapillaris hypoperfusion and the development of PPRCA.To our knowledge, the OCTA features in patients with PPRCA has not been described previously in the literature, and this case report might provide preliminary information regarding the pathophysiology of PPRCA and improve our understanding of the nature of this disease.

## Conclusions

In summary, we reported a Chinese female case of PPRCA and the multimodal imaging of this patient. To the best of our knowledge, this is the first case to report the OCTA features of a PPRCA patient.

## Abbreviations

BCVA: Best corrected visual acuity
ERG: Electroretinogram
FA: Fluorescein angiography
ICGA: Indocyanine green angiography
OCTA: Optical coherence tomography angiography
PPRCA: Pigmented paravenous retinochoroidal atrophy
SD-OCT: Spectral domain optical coherence tomography
